# Self-assembled nanoparticles based on modified cationic dipeptides and DNA: novel systems for gene delivery

**DOI:** 10.1186/1477-3155-11-18

**Published:** 2013-06-21

**Authors:** Jiban J Panda, Aditi Varshney, Virander S Chauhan

**Affiliations:** 1 International Centre for Genetic Engineering and Biotechnology, New Delhi, India; 2 Institute of Liver and Biliary Sciences, New Delhi, India

**Keywords:** Cationic, Dipeptide, Plasmid, Delivery, Nanoparticle

## Abstract

**Background:**

Gene therapy is most effective when delivery is both efficient and safe. However, it has often proven difficult to find a balance between efficiency and safety in case of viral or polymeric vectors for gene therapy. Peptide based delivery systems may be attractive alternatives but their relative instability to proteolysis is a major concern in realizing their potential application in biomedical sciences. In this work we report gene delivery potential of nanoparticles (Nps) synthesized from cationic dipeptides containing a non-protein amino acid α, β-dehydrophenylalanine (∆Phe) residue.

**Methods:**

Dipeptides were synthesized using solution phase peptide synthesis method. Nps were formed using self-assembly. Nps were characterized using light scattering, electron microscopy. Transfection efficiency was tested in hepatocellular carcinoma (HuH 7) cells.

**Results:**

The cationic dipeptides condensed plasmid DNA into discrete vesicular nanostructures. Dipeptide Nps are non-cytotoxic, protected the condensed DNAs from enzymatic degradation and ferried them successfully inside different types of cells. GFP encoding plasmid DNA loaded dipeptide Nps showed positive transfection and gene expression in HuH 7 cells.

**Conclusions:**

The cationic dipeptide Nps can successfully deliver DNA without exerting any cytotoxic effect. Owing to their simple dipeptide origin, ease of synthesis, enhanced enzymatic stability as well unmatched biocompatibility, these could be successfully developed as vehicles for effective gene therapy.

## Background

Gene therapy continues to hold great promise in the field of medicine. Replacing defective genes with functional genes offers many exciting possibilities for treating dreaded diseases such as cancers, autoimmune disorders and neurodegenerative diseases [1]. Efficient gene delivery, fundamental to the success of gene therapy remains a complex process with many possible rate-limiting steps. The most serious intracellular barriers are lysosomal degradation, nucleolytic degradation in the cytosol and inefficient delivery to the nucleus [2-4], whereas, extracellular barriers include nucleolytic degradation in the serum, recognition by the reticulo-endothelial system and nonspecific delivery [5]. Gene delivery in humans requires carriers that will transfer DNA into the nuclei of target cells. These carriers must be efficient in transfection, safe for human use, protect DNA from degradation before arriving at the target cell and possibly hold targeting qualities for the specific delivery of gene to required cells or tissue.

Most early applications of gene therapy were concentrated around viral vectors for efficient gene delivery. However, many of these vectors raised undesirable immune responses, hindering their therapeutic effect [6]. Synthetic gene delivery methods surpassed viral vectors in many ways, such as low cytotoxicity, reduced ability to provoke an immune response, ease of manufacturing and scale up and high adaptability. Over the years many chemical synthetic approaches using polymers with ability to accommodate larger DNA molecules compared to viral vectors, have been developed. Nanoparticles (Nps) loaded with plasmid DNA may serve as efficient sustained release gene delivery systems due to their rapid escape from the degradative endo-lysosomal compartment to the cytoplasmic compartment [7]. Successful oligonucleotide delivery to HeLa cells using anionic liposomes [8], lipid-derived Nps that carry oligonucleotides either in their core or *via* covalent attachment have been reported to have significant efficacy *in vivo* and *in vitro*[9]. Many research groups have used quantum dots [10], magnetic [11] and gold Nps [12,13] as well as carbon nanotubes [14] and enabled successful siRNA/antisense delivery [10,14]. Much attention was given to the biodegradable and biocompatible poly PLGA polymers [15]. Polycationic polymer based Nps as non-viral gene delivery vectors, have also been developed and polyethylenimines is currently the most popular polymer used to deliver genes into various cell types, including neurons. Polyethylenimines is able to condense genes into small Nps and protect the DNA from degradation by nucleases. Polymeric complexes such as PLGA– polyethylenimines Nps have been demonstrated as new delivery systems to carry genes to the lung epithelium [16]. Cationic bovine serum albumin conjugated with poly(ethyleneglycol)–poly(lactide) nanoparticle, was also developed as a promising brain drug delivery carrier with low toxicity [17].

We have been working with very short peptides that self-assemble to form different kind of Nps. Here we report DNA condensation and cell delivery potential of cationic dipeptide Nps (CdNps) such as Arg-∆Phe and Lys-∆Phe. The cationic dipeptides were non-cytotoxic and could condense different plasmid DNAs into discrete Nps enhancing their cellular uptake and delivery, and may represent a novel platform that can be further developed for DNA delivery into cells of different kinds.

## Methods

### Materials

Materials used in this study are: THF, NMM, DMF, piperidine, DIPCDI, IBCF, TIPS, TFA, HFIP, phenol, DL-threo-phenylserine, MTT (Sigma-Aldrich, Munich, Germany) Boc-Arg(Mtr)-OH, Boc-Lys(Boc)-OH (Novabiochem, Merck, Darmstadt, Germany) sodium acetate, ethyl acetate, acetonitrile (Spectrochem Pvt Ltd, Mumbai, India), anhydrous sodium sulfate, citric acid (Merck, Munich, Germany), human ovarian cancer (HeLa), hepatocellular carcinoma (HuH-7) and human dermal fibroblast (L929) cells were obtained from American Type Culture Collection (ATCC, Manassas, VA). RPMI-1640, DMEM, NHS-modified Alexa-488 (Invitrogen, Life Technologies Corp. NY, USA). Plasmids used in the study were generous gifts from Dr Reddy’s laboratory, ICGEB, India. Except for method 2.8 in which plasmids of different lengths [in base pairs (bp)] were used and the transfection experiment where EGFP plasmid was used, all other studies were carried out with pgem plasmid DNA.

### Peptide synthesis

Dipeptides were synthesized at a scale ranging from 10–30 mM by procedures described earlier [18,19] and as provided in the Additional file 1: Information S1.

### Assembly of dipeptides

Assembly of dipeptides into Nps in water was done from their HFIP stock solutions at a concentration of 2 mg/ml. Briefly, 2 mg of cationic peptides were first dissolved in 20 μl of HFIP and then diluted with 1 ml of filtered MilliQ water to form self-assembled Nps.

### Dynamic Light Scattering (DLS) studies

Light scattering studies were performed in Photocor complex (Photocor, Moscow, Russia) using multiple tau digital correlator. For DLS measurement samples were prepared in dust free environment. Experiments were carried out at an angle of 90° using 632 nm laser at room temperature. Data represent mean of three different sets.

### Loading DNA in preformed peptide Nps

Arg-∆Phe and Lys-∆Phe, Nps (2 mg/ml) were prepared as described above and were titrated with increasing concentrations of plasmid DNA (25, 37.5, 50, 62.5, 75, 100 μg) and analyzed using DLS to determine the optimum DNA concentration needed to form compact nanostructures.

### Determination of entrapment efficiency of DNA in Arg-∆Phe and Lys-∆Phe Nps

Plasmid DNA was first labeled with Pt-conjugated Alexa-488 dye using Ulysis DNA-labeling kit (Sigma-Aldrich, Munich, Germany) and then added to Nps (100 μg of plasmid DNA : 1 mg/ml of peptide Nps). Samples were incubated for 30–60 min, filtered through a filter plate with 10 kDa membrane cut-off (Millipore, Corp., Billerica, MA, USA) by centrifuging at 2800 rpm for 20 min at 25°C. Filtrates were analysed spectrophotometerically to measure the amount of free DNA and quantified for percentage entrapment of the nucleotides in Nps as described above by using the following formula:

$$\begin{array}{l} \text{Percentage}\;\text{entrapment}=[\left\{\left(\text{Initial}\;\text{absorbance}\;\text{of}\;\text{the}\;\text{test}-\text{Absorbance}\;\text{of}\;\text{the}\;\text{test}\;\text{filtrate}\right)/\text{Initial}\;\text{absorbance}\;\text{of}\;\text{test})\right\}\\ \;\;\;x100]–\left[\left\{\left(\text{Initial}\;\text{absorbance}\;\text{of}\;\text{control}-\text{Absorbance}\;\text{of}\;\text{filtrate}\right)/\text{Initial}\;\text{absorbance}\;\text{of}\;\text{control}\right\}\;x\;100\right] \end{array}$$.

### Formation of DNA-peptide Nps by Arg-∆Phe and Lys-∆Phe

For making DNA-peptide Nps, 100 μg of plasmid DNA was titrated with increasing amounts of peptides (100, 500, 1000, 2000 μg) and analyzed using DLS.

### Effect of plasmid DNA size on their interaction with Arg-∆Phe and Lys-∆Phe

Peptides (2 mg/ml) were incubated with 100 μg of plasmids with varying sizes for 30 min. The DNA-peptide Nps so obtained were analyzed using DLS and electron microscopy.

### Transmission Electron Microscopy (TEM)

DNA-peptide Nps were adsorbed on a 300 mesh copper grid with carbon coated formvar support and stained with 1% uranyl acetate and viewed under transmission electron microscope operating at an accelerated voltage of 120 kV (Tecnai 12 BioTWIN, FEI Netherlands). Photomicrographs were digitally recorded using a Megaview II (SIS, Germany) digital camera. Image analysis was carried out using Analysis II (Megaview, SIS, Germany) and ImageJ (http://rsb.info.nih.gov/ij/) software packages.

### Localization of DNA in DNA-peptide Nps

In order to determine the exact location of plasmid DNA in DNA-peptide Nps. plasmid DNA was first complexed with platinum using ULYSIS DNA labeling kit and then incubated with Arg-∆Phe (as better Nps were obtained with Arg-∆Phe) for 30 min. Nps so formed were analyzed under TEM.

### Cellular uptake of DNA-peptide Nps

For estimating the cellular uptake of DNA-peptide Nps, plasmid DNA and peptide Nps were labeled with Alexa-488 using ULYSIS DNA labeling kit (Sigma-Aldrich, Munich, Germany) and NHS-modified Alexa-588 dyes (Invitrogen, Life Technologies Corp. NY, USA) respectively. DNA-peptide Nps were prepared as described above. HeLa cells were first cultured in tissue culture dishes in RPMI-1640 media, trypsinized and seeded in 6 well plates (1 x 10^5^ cells/well). After 12 hrs of culture, cells were incubated with plasmid DNA, peptide Nps, DNA-peptide Nps for 18 hrs, washed with serum and phenol red free media and imaged under fluorescence microscope.

### Nuclear localization of DNA-peptide Nps

HeLa cells were seeded on cover slips placed in 6-well plates, allowed to grow for 12 hrs for cell adherence and spreading and subsequently incubated with DNA peptide Nps carrying Alexa-488 labeled DNA for 12 hrs. After this, media was removed, cells were treated with DAPI for nucleus staining, washed and visualized under fluorescent and bright-field microscopes.

### Agarose gel electrophoresis of DNA peptide Nps

Complex formation between Nps and plasmid DNA was analyzed by 1% agarose gel electrophoresis. Free plasmid DNA (4 μg) and DNA peptide Nps complexes with peptide/DNA ratio of 20/1 (4 μg DNA/80 μg of peptide in 40 μl nuclease free water) were run on a 1% (w/v) agarose gel with TBE buffer (89 mmol/l Tris, 89 mmol/l boric acid, and 2 mmol/l EDTA pH 8.0) at 75 V for 90 minutes. DNA was visualized by staining gels with ethidium bromide (0.5 g/l) and images were acquired using an UV trans-illuminator (Vilber, Lourmat, France). Stability of DNA peptide Nps towards enzymatic degradation was determined by incubating plasmid DNA (4 μg) and DNA peptide Nps complexes with peptide/DNA ratio of 20/1 (4 μg DNA/80 μg of peptide in 40 μl nuclease free water) with 5 μl of DNase I solution (10 μg/ml in DNase/Mg^2+^ digestion buffer, which consisted of 50 mM Tris–HCl, pH 7.6, and 10 mM MgCl_2_) each at 37°C for 30 min, and degradation of plasmid DNA was analyzed by 1% agarose gel electrophoresis.

### Cytotoxicity of DNA-peptide Nps

HeLa and L929 cells were cultured in RPMI-1640 and DMEM supplemented with 10% FBS, and maintained on TCTP plates at 37°C in a humified atmosphere containing 5% CO_2,_ till 70-80% confluency. Cells were then harvested by trypsinization, washed and resuspended in RPMI-1640/DMEM medium (supplemented with 10% FBS). Live cells were counted by trypan blue dye exclusion test using a hemocytometer, diluted in completed media, seeded in 96 well plates (2 x 10^4^ cells/well). After 12 hrs, cells were incubated with 20 μl each of dipeptide and DNA-dipeptide Nps (2 mg/ml) for a period of 24 hrs. As a control, cells were incubated with 20 μl of PBS. MTT assay was used to determine the cell viability of treated cells. The percentage viability was calculated using the equation given below:

Percentage viability of cells = [(Absorbance of MTT formazan produced by cells grown in presence of nps /Absorbance of MTT formazan produced by cells grown on TCTP control plate)] × 100.

### In vitro transfection efficiency of DNA peptide Nps

HuH-7 cells were cultured in DMEM supplemented with 10% FBS and 1% pencillin-streptomycin and maintained on TCTP plates at 37°C in a humified atmosphere containing 5% CO_2,_ till 70-80% confluency. 24 h prior to transfection cells were seeded in 6 well plates (2 x 10^5^). DNA-Nps were prepared by incubating 4 μg of EGFP encoding plasmid DNA with 80 μg each of dipeptides Lys-∆Phe and Arg-∆Phe in 40 μl of nuclease free water. Nps so formed were visualized under TEM to determine particle size and morphology as described above. On the day of transfection, the culture medium in each well was replaced with 1 ml of complete medium containing 1 μg of DNA loaded in DNA-peptide Nps at (1: 200 DNA to peptide ratio) and 1 μg of DNA in lipofectamine. The cells were then incubated for 48 hrs, washed to remove excess Nps and DNA and analysed using fluorescence microscopy for GFP expression.

### Statistical analysis

The results are presented as the mean ± SD calculated over at least three data points.

## Results and discussion

Cationic polymer based systems have been widely used to condense DNA inside their core to protect it from intra-cellular nucleases and achieve efficient delivery [16,20]. Arg-∆Phe and Lys-∆Phe, due to the presence of positive charges on them, were used to condense plasmid DNA of various sizes into discrete Nps. Plasmid DNAs were loaded on to dipeptide Nps either by incubating them with preformed Nps or by the formation of DNA-nanoparticle complexes by slow mixing of the aqueous solution of cationic dipeptides and plasmids.

**Figure 1:**
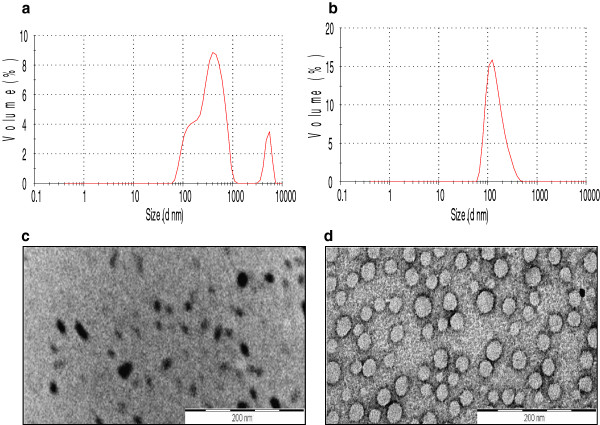
**Nps formed by Lys**-**ΔPhe and Arg**-**ΔPhe at a concentration of 2 mg**/**ml.** Hydrodynamic diameter measurements of Lys-∆Phe Nps at a concentration of 2 mg/ml **(a)** and Arg-∆Phe at 2 mg/ml **(b)**. TEM images of Lys-∆Phe **(c)** and Arg-∆Phe Nps **(d)**. Scale bar 200 nm.

Light scattering studies demonstrated that at a concentration of 2 mg/ml (in HFIP-water), Arg-∆Phe self-assembled into Nps with mean solution hydrodynamic radius (Rh) of 150 nm and a polydispersity index of 0.1. Nps formed by Lys-∆Phe at this concentration exhibited broad size distribution with a polydispersity of 0.38 (Figure 1a and b). In HFIP-water, the dipeptide formed particles with two major populations having mean Rh of 200 nm and 400 nm. TEM showed that Lys-∆Phe, at a concentration of 2 mg/ml self-assembled to form structures of irregular boundaries with a mean diameter of 50 nm (Figure 1c). These particles were different from the vesicular structures (370 nm) formed by the dipeptide at a concentration of 10 mg/ml [18], pointing towards concentration dependent assembly. However, Arg-∆Phe at a concentration of 2 mg/ml showed better assembly than Lys-∆Phe and formed well defined spherical Nps with an average size of 40 nm (Figure 1d). This is perhaps due to the fact that in arginine, the guanidinium group remains positively charged in neutral, acidic and even in low basic conditions promoting enhanced charge based interactions and hence self-assembly.

**Figure 2:**
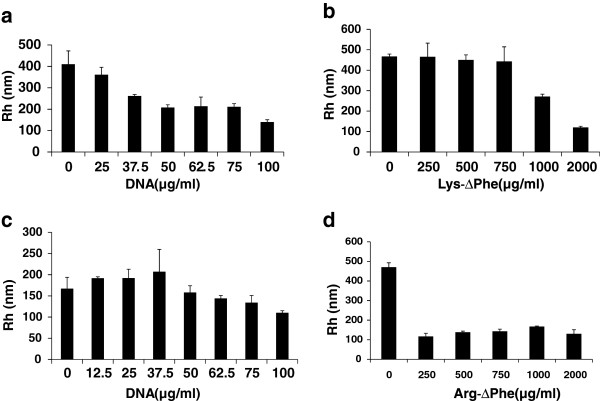
**DLS study showing Np formation by Lys**-**ΔPhe and Arg**-**ΔPhe with DNA. (a)** Lys-∆Phe Nps + DNA. **(b)** DNA + Lys-∆Phe. **(c)** Arg-∆Phe Nps + DNA. **(d)** DNA + Arg-∆Phe. Peptide Nps interacted with DNA leading to change in their overall size (Rh). Data expressed as mean ± SD (n = 3).

Light scattering technique was used to study the interaction of plasmid DNA with dipeptide Nps as well as to determine the optimum concentration of DNA needed for making compact and well defined Nps. Lys-∆Phe on its own formed Nps of Rh of 400 nm. Upon addition of increasing concentration of DNA [(25–100 μg) to 2 mg/ml of Nps], the Nps showed a reduction in their particle size. At a concentration of 100 μg of DNA, particles were further compacted and showed an average Rh of ~140 nm (Figure 2a). However, further addition of DNA did not lead to any significant change in particle size. When Arg-∆Phe Nps (2 mg/ml) were incubated with increasing concentrations of DNA (25–100 μg), their Rh decreased from approximately 170 nm to 110 nm (Figure 2c). The observed reduction in particle size of dipeptide Nps upon DNA addition could be due to charge-charge interactions of positively charged peptide Nps with negatively charged DNAs (Figure 2). Many earlier observations have also shown that cationic Nps bearing positive charge interact with DNA leading to DNA compaction and reduction in particle size [21-23].

From the above study, the optimum peptide to DNA ratio needed to form suitably compacted DNA-peptide Nps was found to be 20:1 (w/w). Entrapment efficiency determined from centrifugation experiments of the fluorescence labeled DNA showed almost 95% entrapment in both Arg-∆Phe and Lys-∆Phe Nps at this peptide to DNA ratio.

Cationic polymers condense DNA to yield compact Nps with well defined sizes [16,24]. Nanoparticle formation was also observed when the cationic dipeptides were added to plasmid DNA. While plasmid DNA alone in water (100 μg/ml) formed irregular structures (no regular structure was found under TEM) with a mean Rh of 450 nm, it condensed into smaller and monodispersed Nps with the addition of increasing concentration of the cationic peptides. Nps with an average Rh of 120 nm were formed by the addition of 2 mg of Lys-∆Phe (Figure 2b) to the plasmid DNA. Thus, in this case nanoparticle formation occurred by the condensation of plasmid DNA in presence of cationic dipeptides and size of the resultant Nps depended on the amount of peptide added. Similar observation was seen in case of BSA-plasmid DNA polyplexes, where an increase in BSA/plasmid DNA mass ratio led to decrease in overall particle size of the resultant polyplexes [25]. Likewise, addition of increasing concentration Arg-∆Phe also led to DNA condensation and decrease in overall particle size (Figure 2d). It was also observed that Arg-∆Phe led to DNA condensation at lower concentration as compared to Lys-∆Phe (250 μg of Arg-∆Phe vs 2000 μg Lys-∆Phe for same amount of DNA). Similar observation has been shown earlier where better DNA condensation was obtained with ariginine homopeptides than lysine ones [26]. This could be explained by the difference in the nature of electrostatic interactions and DNA binding capacity between lysine and arginine residues [27]. Where there is only one amine group in the lysine side chain guanidinium side chain of arginine has three amine groups. This promotes zwitterion hydrogen bonding of arginine with the phosphate group as well as guanine base of DNA, providing high binding strength and assembly than lysine [28].

**Figure 3:**
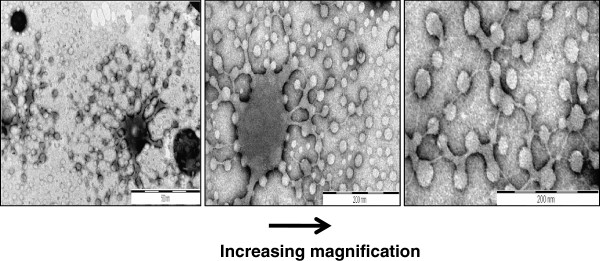
**Condensation of DNA into well defined vesicular Nps by Arg-∆Phe (it is clearly visible how densely stained blotch of DNA gets compacted into vesicular structures with defined boundary and size).**

Nps with a mean Rh of 117 nm were formed when 250 μg of Arg-∆Phe was added to 100 μg of DNA in 1 ml of water. A further addition of peptides did not cause any considerable change in particle size. TEM images further supported the idea of DNA condensation by Arg-∆Phe and showed initial formation of large, densely stained (due to the presence of negatively charged DNA) aggregates, which then rearranged into well defined spherical structures with time. The Nps were highly homogenous in nature with mean diameter ranging from 20–30 nm (Figure 3) a size suitable for endocytotic cellular uptake [29].

Effect of DNA size on the formation of DNA-peptide Nps was determined by incubating cationic dipeptides with plasmids of various sizes. Lys-∆Phe formed particles with mean Rh of 70 nm with pl, 117 nm with pgem, 165 nm with dh, 171 with md, and 112 with sod (Additional file 2: Figure S1a). Similarly, Arg-∆Phe formed particles with Rh of 103 nm with pl, 134 nm with pgem, 280 nm with dh, 479 nm with md and 280 nm with sod (Additional file 2: Figure S1b). Thus it was observed that there was no correlation between the size of DNA-peptide Nps with plasmid length (in base pairs) (Additional file 2: Figure S1).

**Figure 4:**
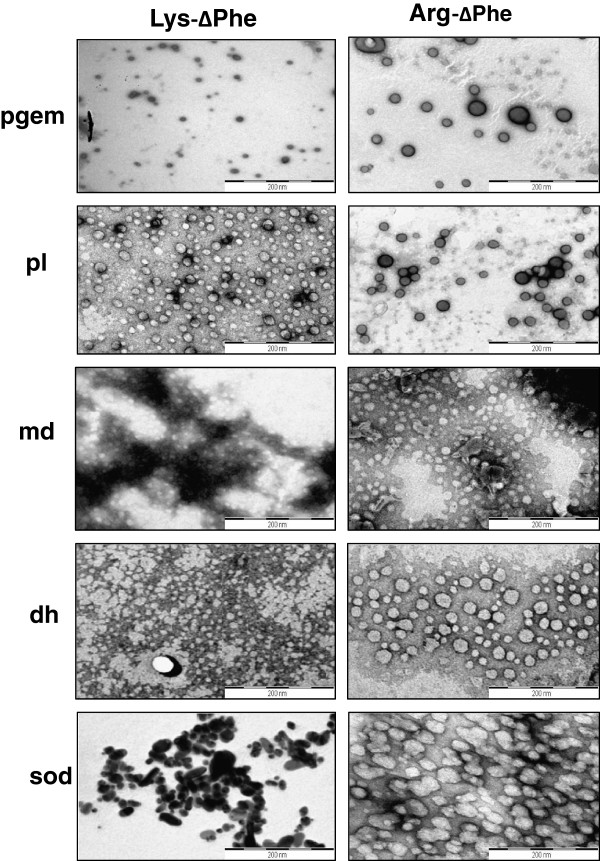
**Nps prepared by condensing different plasmid DNAs with Lys**-**ΔPhe and Arg-∆Phe.** Scale bar 200 nm.

The morphology of Nps formed by combination of different plasmid DNAs with Lys-∆Phe and Arg-∆Phe was determined under electron microscopy. Lys-∆Phe condensed plasmid DNA pl into well defined vesicular nanostructures (Figure 4) with mean size of 30 nm. The peptide formed micellar structures with md and dh DNAs with average particle size of approximately 20 nm. With sod plasmid DNA, the peptide formed densely stained ellipsoid like structures with mean size of 50 nm. Whereas, combination of Lys-∆Phe with pgem DNA resulted in the formation of roughly vesicular nanostructures with mean diameter of 100 nm. Similarly, Arg-∆Phe formed regular and well defined vesiclular nanostructures with plasmid DNAs of pgem, md, pl and md having mean diameters of 40, 30 and 42 and 50 nm respectively. However, with sod plasmid DNA, Arg-∆Phe formed nanostructures with irregular boundaries with average size of ~60 nm. Thus, both peptides formed Nps with variable morphology with different plasmid DNAs. Earlier studies have also demonstrated that multivalent cations such as polyamines, positively charged polymers, and peptides promote condensation of DNA to Nps that appear as rods, toroids, or spheroids under electron microscope [26,30,31].

It was also observed that in most cases Nps formed by the combination of Arg-∆Phe with plasmid DNAs were more regular as compared to those formed by the Lys-∆Phe and plasmid DNAs (Figure 4). This correlates with our DLS results that showed better DNA condensation and assembly with Arg-∆Phe as compared to Lys-∆Phe.

**Figure 5:**
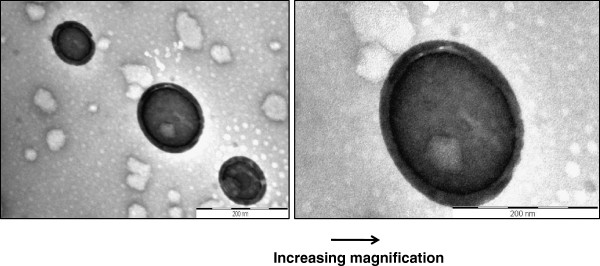
**TEM images showing location of DNA inside Arg-∆Phe**-**DNA Nps (densely stained structures are the peptide Nps which entrapped DNA).** DNA is labeled with electron dense Pt for aiding visualization under TEM. High magnification images clearly indicate the presence of DNA inside nanoparticle core. Scale bar 200 nm.

In order to locate DNA in dipeptide Nps, plasmid DNA was labeled with Pt-complex; Pt being electron dense can aid in locating the labeled DNA inside DNA-peptide Nps in electron microscopy. Electron micrographs of Pt-DNA-dipeptide Nps showed densely stained Pt-DNA-complexes lying inside normal vesicular structures. High resolution micrographs demonstrated core-shell like structure with dark ring like DNA inside nanoparticle core (Figure 5).

**Figure 6:**
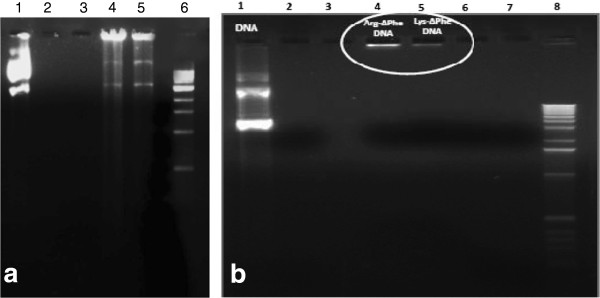
**Gel electrophoresis of DNA**-**peptide Nps. (a)** Gel electrophoresis showing complexation of Arg-∆Phe and Lys-∆Phe with plasmid DNA. Lane 1 contains plasmid DNA, 2 and 3 lanes are only dipeptides, lanes 4, 5 show Arg-∆Phe-DNA Nps and Lys-∆Phe-DNA Nps respectively and lane 6 contains DNA ladder. **(b)** Gel electrophoresis showing resistance of DNA-peptide Nps towards DNase I treatment. Lane 1 contains plasmid DNA, lanes 4, 5 show Arg-∆Phe DNA Nps and Lys-∆Phe DNA Nps respectively and lane 8 contains DNA ladder. Nanoparticle formulations with peptide/DNA ratio of 20/1 and free DNA (4 μg) were separately incubated with 5 μl of DNase I solution (10 μg/ml in DNase/Mg^2+^ digestion buffer, which consisted of 50 mM Tris–HCl, pH 7.6, and 10 mM MgCl_2_) at 37°C for 30 min, and degradation of plasmid DNA was analyzed by electrophoresis on a 0.8% agarose gel.

Complexation of DNA with dipeptide Nps was further investigated using agarose gel electrophoresis [15,24]. It was observed that DNA was retained around sample pore after being complexed with peptide Nps (Figure 6a). Nps bound to DNA remained positively charged, causing DNA binding to them to have retarded movement into the gel. Similar results and retention in DNA mobility were found in case of genes and DNAs when bound to Nps [15,24].

A major hurdle in gene or siRNA delivery is the degradation of DNAs by intra and inter-cellular nucleases. Hence an ideal gene delivery vehicle should not only facilitate intracellular delivery of loaded DNAs but also protect them from degradation by inter or intra-cellular nucleases [32-34]. In order to test the stability of DNA-peptide Nps, Nps were digested with DNase I and analyzed using agarose gel electrophoresis. Free plasmid DNA was degraded into smaller fragments. However, DNA-peptide Nps digested by DNase I were retained around sample pore (Figure 6b). These results showed that DNA was largely protected from enzymatic degradation in the DNA-dipeptide Nps.

**Figure 7:**
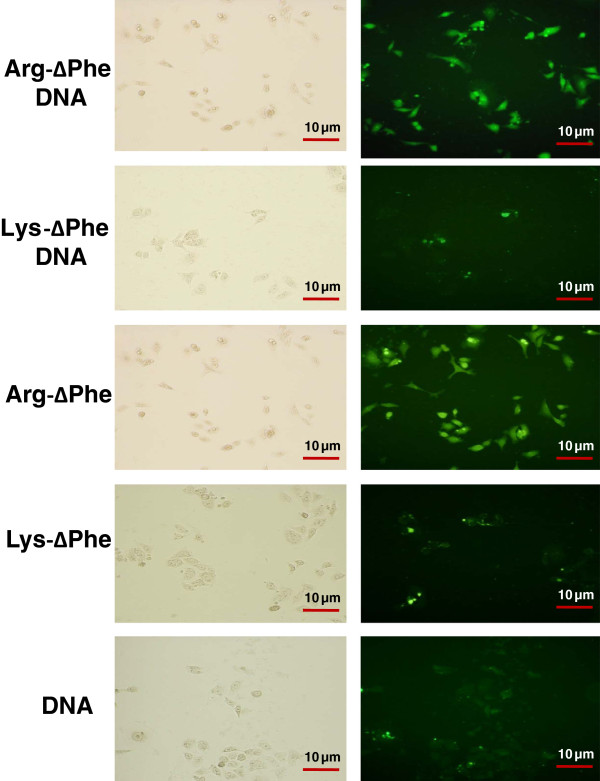
**Optical bright field and fluorescent images showing entry of Alexa**-**488 labeled DNA**-**peptide Nps into HeLa cells.** Magnification: 20X.

Efficacy of the DNA-dipeptide Nps to ferry DNA inside cells was determined in HeLa cells. It was observed that cells could easily take up the DNA-peptide Nps and appeared fluorescent in all four cases. Fluorescence intensity and hence cellular uptake was greater in case of Arg-∆Phe-DNA Nps as compared to Lys-∆Phe-DNA Nps (Figure 7). This may be attributed to better assembly and DNA condensing ability of Arg-∆Phe into discrete and compact nanostructures facilitating their cellular uptake. Similar phenomenon was observed in earlier reports that demonstrated that the cellular uptake of the DNA-peptide complexes and their intracellular state seem to be controlled by the peptide chemistry and homopeptides made of arginine demonstrated higher cellular uptake than their lysine counterparts [26,35]. Cells were also incubated in presence of Alexa-488 labeled free DNA or void peptide Nps as controls. Results revealed that though HeLa cells could easily take-up void peptide Nps they showed very little uptake of free DNA (Figure 7). Thus, it was clearly seen that packaging DNA inside peptide Nps improved their cellular uptake ability and could be instrumental in non-viral mediated gene delivery. Moreover, the degree of nanoparticle uptake by cells correlated directly with their incubation time and was higher for an incubation period of 12 hrs as compared to that of 6 hrs (data not shown).

**Figure 8:**
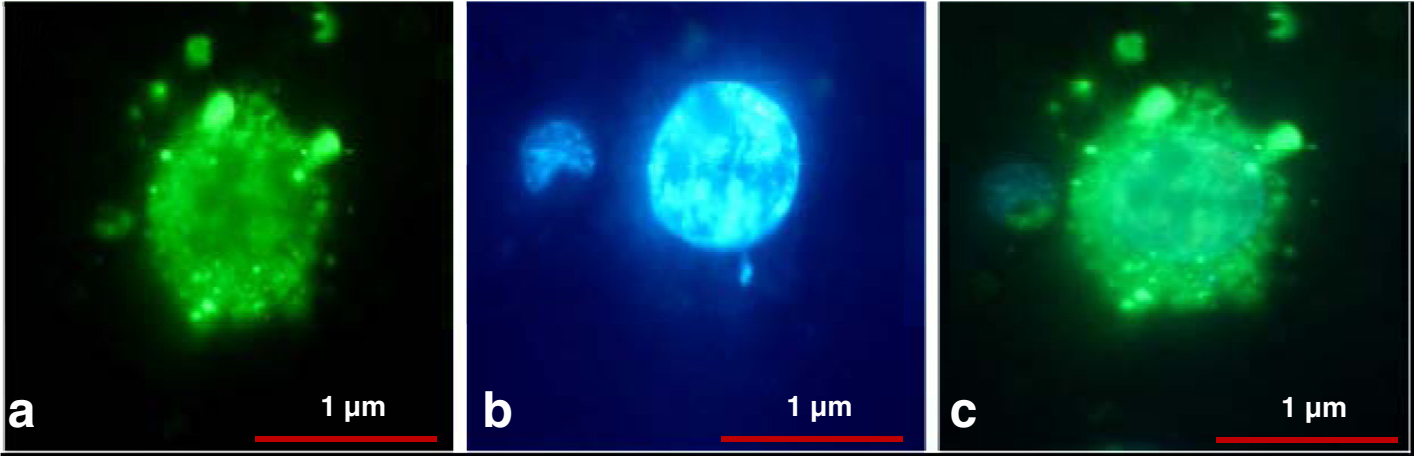
**Localization of DNA loaded Arg-∆Phe Nps in the nucleus of L929 cells. (a)** Fluorescent image showing cellular uptake of Alexa-488 labeled DNA loaded in Arg-∆Phe Nps. **(b)** Image taken under UV light showing nucleus stained with DAPI. **(c)** Merged image showing nuclear uptake of DNA when loaded in the dipeptide Nps. Magnification: 100X.

We next investigated if DNA-peptide Nps were localized in the nucleus of HeLa cells. Cells were incubated with fluorescent-labeled DNA-peptide Nps and imaged under microscope. Nuclei were labeled with DAPI staining. Fluorescent micrographs showed that DNA-peptide Nps were easily taken up by cells and the engulfed DNA was distributed all through the cytoplasm and the nucleus (Figure 8). These results suggest that small cationic peptide based Nps can condense DNA into discrete Nps and can carry them to cellular cytoplasm and nucleus protecting them from enzymatic degradation.

**Figure 9:**
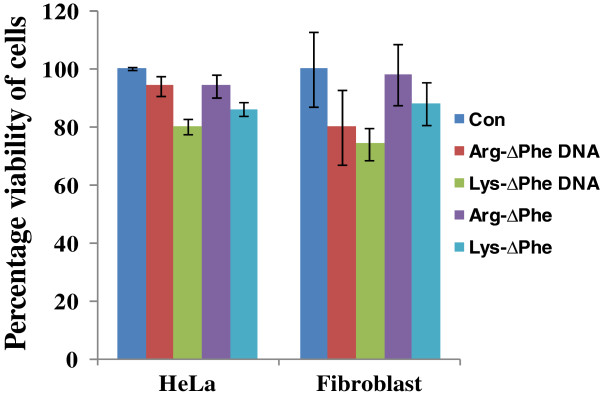
**Percentage viability of cells incubated with DNA peptide Nps determined using MTT assay.** DNA-dipeptide Nps are noncytotoxic. (con: only media control). Data expressed as mean ± SD (n = 3).

Toxicity is a major issue in synthetic polymer based gene delivery [36]. Toxicity of the peptide and DNA-peptide Nps was determined using MTT assay. Results of MTT cell viability assay demonstrated that DNA-peptide Nps were biocompatible and showed no cytotoxicity to cell lines like HeLa and L929 (Figure 9). This is an important characteristic property of Arg-∆Phe and Lys-∆Phe Nps which make them suitable for gene therapeutic applications; several other cationic polymers have been found to be more toxic [37].

**Figure 10:**
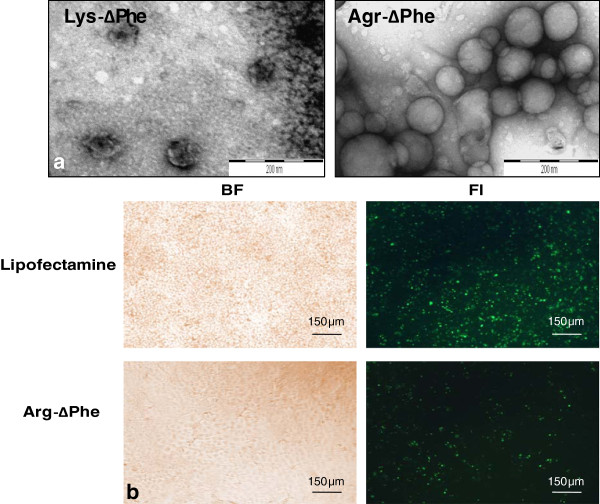
**Expression of EGFP with dipeptide Nps. (a)** TEM images of Nps formed by condensing EGFP plasmid DNA with Lys-∆Phe and Arg-∆Phe. Scale bar 200 nm. **(b)** Fluorescent images showing transfection and EGFP expression in HUH 7 cells by Arg-∆Phe Nps (10 X magnification).

After establishing positive plasmid DNA delivery as well as biocompatibility of DNA-peptide Nps, we next evaluated the transfection efficiency of the Nps. For this the dipeptides were incubated with GFP encoding plasmid DNA (peptide to DNA ratio of 20:1) and analyzed using TEM. Images showed that Nps formed by the combination of Arg-∆Phe with EGFP plasmid DNA were vesicular in nature with mean diameter of 80 nm approximately, whereas those formed by the plasmid DNA and Lys-∆Phe were irregular with no proper shape and size (Figure 10a). These results corroborated with our earlier results carried out with other plasmid DNAs, where Arg-∆Phe demonstrated better DNA condensation and assembly than Lys-∆Phe. Since better Np formation was obtained with Arg-∆Phe, cellular transfection assay for determining EGFP expression was carried out with Arg-∆Phe. To test expression and transfection efficiency of DNA-dipeptide Nps, HuH7 cells were incubated with EGFP encoding plasmid DNA Arg-∆Phe and lipofectamine and analysed using fluorescence microscopy. Images showed positive green fluorescence in both cases depicting successful plasmid DNA delivery and expression by the dipeptide Nps (Figure 10b).

## Conclusions

The work presented here showed that cationic dipeptides containing a chemically modified amino acid, ∆Phe, easily formed discrete nanoparticles with plasmid DNA of different lengths. Dipeptide nanoparticles protected plasmid DNA from enzymatic degradation and easily ferried them inside mammalian cells, both in nucleus and cytoplasm. GFP encoding plasmid DNA loaded Nps showed positive transfection and gene expression in HuH 7 cells. The dipeptides described here are easy to synthesize and also demonstrated no visible cytotoxic effect in tested mammalian cells. To conclude the study brings to light the potency of cationic dipeptide based systems as gene delivery vehicles and certainly adds new dimensions to non-viral based gene delivery.

## Abbreviations

∆Phe: α, β-dehydrophenylalanine; Lys: lysisne; Arg: Arginine; Nps: Nanoparticles; HFIP: 1,1,1,3,3,3-hexa-fluoro-isopropanol; THF: Tetrahydrofuran; NMM: N-methyl morpholine; DMF: Dimethylformamide; DIPCDI: N,N’-Diisopropylcarbodiimide; DLS: Dynamic light scattering; IBCF: Isobutyl chloroformate; TIPS: Triisopropylsilane; TFA: Trifluoroacetic acid; Md: Plasmid DNA of 5.2 kb [Topo 2.1 vector + mondehydroascorbate reductase gene]; Dh: Plasmid DNA of 6.1 kb [pET14b vector + dehydroascorbate reductase gene]; Sod: Plasmid DNA of 5.8 kb [pET14b + superoxide dismutase gene]; Pgem: Plasmid DNA of 5.26 kb [pGreen + Rd2qA gene]; Pl: Plasmid DNA of 3.69 kb [PL12R34H]; HeLa: Human cervical cancer; HuH: Human hepatocellular carcinoma; L929: Human fibroblast; MTT: (3-(4,5-Dimethylthiazol-2-yl)-2,5-diphenyltetrazolium bromide; Pt: Platinum; BSA: Bovine serum albumin; FBS: Fetal calf serum; TCTP: tissue culture treated polystyrene; EDTA: Ethylenediaminetetraacetic acid; TBE: Tris/Borate/EDTA; NHS: N-hydroxysuccinimide; DAPI: 4′,6-diamidino-2-phenylindole; PLGA: poly (D,L-lactide-co-glycolide); SD: standard deviation; nm: nanometer; PBS: phosphate buffered saline; UV: ultra-violet; Bp: base pairs; Kb: kilobase pairs; Rh: hydrodynamic radius; TEM: transmission electron microscopy; EGFP: enhanced green fluorescent protein; kV: kilo volt; Arg-∆Phe: Arginine-α, β-dehydrophenylalanine and Lys-∆Phe; Lysine-α: β-dehydrophenylalanine; Boc: butoxycarbonyl; Mtr: 4-Methoxy-2,3,6-trimethylbenzenesulphonyl.

## Competing interests

Authors declare that they have no competing interests with any organization or body.

## Authors’ contributions

JJP conceived, designed and performed all experiments. AV carried out GFP transfection studies. VSC directed the research. JJP and VSC wrote the manuscript. All authors have read and approved the manuscript.

## Supplementary Material

Additional file 1 **Information S1.** contains details about the synthesis of Arg-∆Phe and Lys-∆Phe dipeptides. Click here for file

Additional file 2: Figure S1 Change in overall size of DNA-peptide Nps when incubated with plasmid DNA of different lengths (in base pairs). (a) Change in size of Arg-∆Phe-DNA Nps. (b) Change in size of Lys-∆Phe-DNA Nps. [Blue bars show plasmid DNA size in base pairs (bp) and red bars show mean Rh of Nps determined through DLS (np size)]. Click here for file
