# Supplementary material for: Self-assembled nanoparticles based on modified cationic dipeptides and DNA: novel systems for gene delivery
Source: J Nanobiotechnology. 2013 Jun 21;11:18. doi: 10.1186/1477-3155-11-18 (PMC3707807; doi:10.1186/1477-3155-11-18)
Supplement: Additional file 1 — Information S1. contains details about the synthesis of Arg-∆Phe and Lys-∆Phe dipeptides. [file 1477-3155-11-18-S1.docx]

**Supplementary information**

**Information S1**

**Synthesis of H-Arg-ΔPhe-OH**

Boc-Arg(Mtr)-OH (Novabiochem) (2.4g, 5mM) was dissolved in dry tetrahydrofuran (Sigma-Aldrich) and the resulting solution stirred in an ice-salt bath at -15°C. N-methyl morpholine (Sigma) (0.65ml, 5mM) was added to the solution followed by isobutyl chloro-formate (Sigma) (0.7ml, 5mM). After 10min, a pre-cooled aqueous solution of DL-threo-β-phenylserine (Sigma-Aldrich) (1g, 5.5mM) and sodium hydroxide (0.22g, 5.5mM) was added and mixture stirred overnight at room temperature. The reaction mixture was concentrated *in vacuo*, acidified with citric acid to pH 3.0 and extracted with ethyl acetate (Spectrochem) (3×20ml). The ethyl acetate layer was washed with water (2×15ml), with saturated sodium chloride (1×20ml), dried over anhydrous sodium sulfate and evaporated to yield Boc-Arg(Mtr)-DL-threo-β-phenylserine as an oily compound (3.2g, ~100%). The compound, Boc-Arg(Mtr)-DL-threo-β-phenylserine, was then mixed with anhydrous sodium acetate (0.53g, 6.5mM) in freshly distilled acetic anhydride (50ml) and stirred for 36hrs at room temperature. The thick slurry obtained was poured over crushed ice and stirred till the oily suspension gave rise to a yellow colored solid. The precipitate was filtered, washed with 5% NaHCO_3_, cold water and dried under vacuum. The resulting azalactone, Boc-Arg(Mtr)-ΔPhe-Azl (2.9g, 4.8mM), was dissolved in methanol, treated with 1.5 equivalents of 1N NaOH solution and stirred at room temperature for 3-4hrs. The mixture was then partially evaporated to remove methanol, acidified with citric acid to pH 3.0 and extracted with ethyl acetate (3×30ml), the combined ethyl acetate extract was washed with water (2×20ml), dried over anhydrous sodium sulphate and evaporated to yield Boc-Arg(Mtr)-ΔPhe-OH (2.9g, 4.5mM) as a white solid. Deprotection at the α-amino group was achieved by treatment with 98% formic acid (30ml) for 3hrs or 50% Trifluoro-acetic acid (TFA):Dichloromethane (DCM) for 1hr at room temperature. The reaction mixture was evaporated to dryness and the residue was precipitated with anhydrous diethyl ether (50ml). The resulting precipitate was filtered, washed several times with dry ether and subsequently lyophilized from 10% acetic acid-water (20ml) to yield the final compound H-Arg-ΔPhe-OH as white powder.

The peptide was purified on a preparative reverse phase C_18_ column (Deltapak, C_18_, 15μ, I.D. 300×19mm) using acetonitrile-water linear gradient 5- 95% acetonitrile [0.1% trifluoroacetic acid (TFA)] /water (0.1% TFA) at a flow rate of 4ml/min over 55min. The purified peptide was reinjected into an analytical reverse phase C_18_ column (Phenomenex, C_18_, 5μ, I.D. 250×4.6mm) using a acetonitrile-water linear gradient 5-95% acetonitrile (0.1%TFA)/water (0.1% TFA) at a flow rate of 1ml/min over 55 min and was found to be 98% pure ( Sup Table 1 ). The purified peptide was analyzed by mass spectroscopy (Applied Biosystems QStar (Q-TOF)).

Other dipeptide H-Lys-ΔPhe-OH was synthesized and purified using the same procedure described above, starting from Boc-Lys (Boc)-OH.
