# Supplementary figures and images for: Self-assembled nanoparticles based on modified cationic dipeptides and DNA: novel systems for gene delivery
Source: J Nanobiotechnology. 2013 Jun 21;11:18. doi: 10.1186/1477-3155-11-18 (PMC3707807; doi:10.1186/1477-3155-11-18)

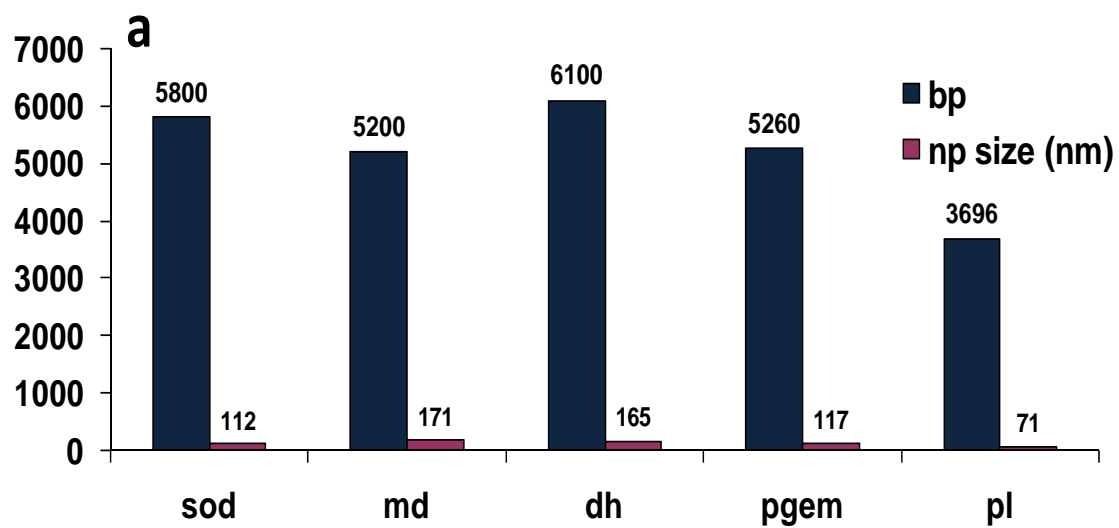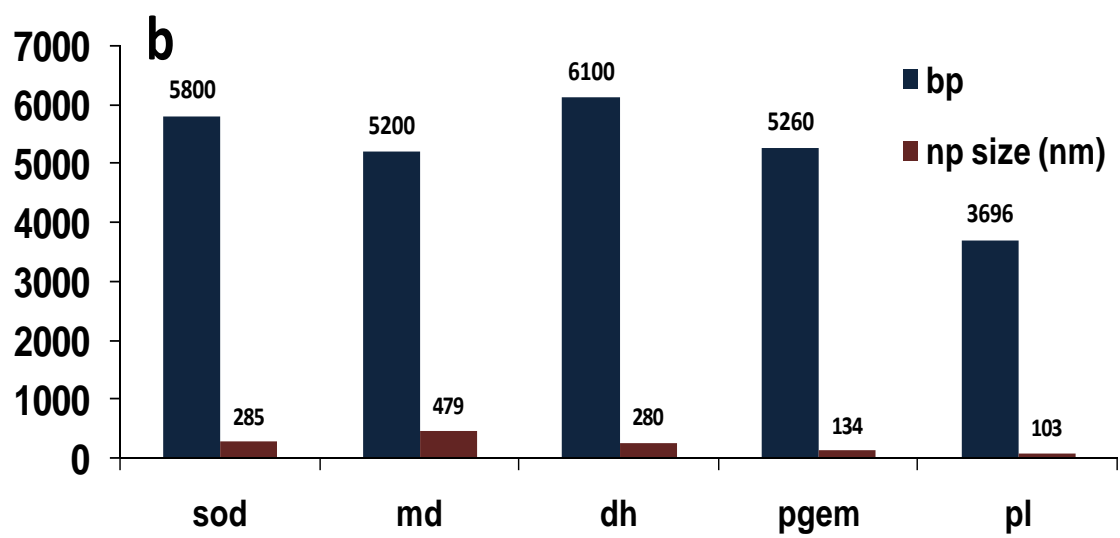

Supplement: Additional file 2: Figure S1 — Change in overall size of DNA-peptide Nps when incubated with plasmid DNA of different lengths (in base pairs). (a) Change in size of Arg-∆Phe-DNA Nps. (b) Change in size of Lys-∆Phe-DNA Nps. [Blue bars show plasmid DNA size in base pairs (bp) and red bars show mean Rh of Nps determined through DLS (np size)]. [file 1477-3155-11-18-S2.pdf]
